# Eukaryotic and Prokaryotic Phytochelatin Synthases Differ Less in Functional Terms Than Previously Thought: A Comparative Analysis of *Marchantia polymorpha* and *Geitlerinema* sp. PCC 7407

**DOI:** 10.3390/plants9070914

**Published:** 2020-07-20

**Authors:** Erika Bellini, Claudio Varotto, Marco Borsò, Lorenza Rugnini, Laura Bruno, Luigi Sanità di Toppi

**Affiliations:** 1 Department of Biology, University of Pisa, 56126 Pisa, Italy; erika.bellini@biologia.unipi.it; 2 Department of Biodiversity and Molecular Ecology, Research and Innovation Centre, Fondazione Edmund Mach, 38100 San Michele all’Adige (TN), Italy; claudio.varotto@fmach.it; 3 Department of Surgery, Medical, Molecular, and Critical Area Pathology, University of Pisa, 56124 Pisa, Italy; marco.borso@student.unisi.it; 4 Department of Biology, University of Rome “Tor Vergata”, 00133 Roma, Italy; lorenza.rugnini@uniroma2.it (L.R.); laura.bruno@uniroma2.it (L.B.)

**Keywords:** bryophytes, cadmium, cyanobacteria, glutathione, heavy metals, liverworts, phytochelatins, phytochelatin synthase

## Abstract

This paper reports functional studies on the enzyme phytochelatin synthase in the liverwort *Marchantia polymorpha* and the cyanobacterium *Geitlerinema* sp. strain PCC 7407. In vitro activity assays in control samples (cadmium-untreated) showed that phytochelatin synthase was constitutively expressed in both organisms. In the presence of 100 µM cadmium, in both the liverwort and the cyanobacterium, the enzyme was promptly activated in vitro, and produced phytochelatins up to the oligomer PC_4_. Likewise, *in vivo* exposure to 10–36 µM cadmium for 6-120 h induced in both organisms phytochelatin synthesis up to PC_4_. Furthermore, the glutathione (GSH) levels in *M. polymorpha* were constitutively low (compared with the average content in higher plants), but increased considerably under cadmium stress. Conversely, the GSH levels in *Geitlerinema* sp. PCC 7407 were constitutively high, but were halved under metal treatments. At odds with former papers, our results demonstrate that, as in *M. polymorpha* and other plants, the cyanobacterial phytochelatin synthase exposed to cadmium possesses manifest transpeptidasic activity, being able to synthesize phytochelatins with a degree of oligomerization higher than PC_2_. Therefore, prokaryotic and eukaryotic phytochelatin synthases differ less in functional terms than previously thought.

## 1. Introduction

Phytochelatin synthase (PCS) is a γ-glutamylcysteine-dipeptidyl-(trans)peptidase (EC 2.3.2.15) belonging to the “Clan CA” of papain-like cysteine proteases [1,2]. In eukaryotes, PCSs are expressed in the vast majority of land plants [2,3,4] and algal groups [5,6,7], as well as in a number of Fungi (including lichens), Animalia, Amoebozoa, Stramenopila, Alveolata, Rhizaria, and Excavata [2,3,8,9,10].

Eukaryotic PCSs, particularly plant PCSs, display manifest transpeptidasic activity by catalyzing the cytosolic synthesis of phytochelatin (PCn) thiol-oligopeptides, whose general structure is (γ-glutamate–cysteine)*_n_*-glycine, with *n* ranging from 2 to 5; for this reason, PCn oligomers are named PC_2_, PC_3_, PC_4_, and PC_5_. The cysteine thiol-groups enable PCn to chelate several metal(loid)s and segregate them in the vacuo-lysosomal compartments [2,11], thus drastically reducing the metal(loid) toxicity. The transpeptidasic activity of PCS is manifested when the enzyme’s catalytic site tightly binds complexes between glutathione in reduced form (GSH) or its direct thiol-derivatives, and metal(loid)s such as cadmium (Cd), lead (Pb), mercury (Hg), arsenic (As), copper (Cu), zinc (Zn), and iron (Fe) [3,11]. 

In prokaryotes, PCS enzymes with moderate sequence homologies to those of plants have been found in some cyanobacteria and β- and γ-proteobacteria ([3]; and references therein). So far, the very limited number of studies conducted on cyanobacterial PCS—in particular on *Nostoc* sp. PCC 7120 (sin.: *Anabaena* sp. PCC 7120)—suggest that this enzyme is a “half-size” protein with good homology to the *N*-terminal domain of eukaryotic PCS [12]. However, unlike PCS from plants and other eukaryotes, in cyanobacteria the enzyme only acts as a peptidase, thus regulating the catabolism of GSH-conjugates, by cleaving the glycine residue from GSH [13,14]. In fact, no differences in cyanobacterial PCS activity under metal stress have ever been detected when compared with untreated controls [12,14,15]. Further work showed that the PCS from *Nostoc* sp. PCC 7120 displays extremely weak transpeptidasic activity, regardless of whether Cd is present or not, resulting in the production of very low levels of PC_2_, but no PCn with higher degrees of oligomerization [12]. Interestingly, phylogenetic analysis supports a cyanobacterial ancestry of eukaryotic PCS genes; yet, phylogenetic relationships of PCS and prokaryotic PCS-like genes are not congruent with the topology of current taxonomic trees, thus suggesting multiple events of horizontal gene transfer [16]. Indeed, considering that cyanobacteria have a robust evolutionary link with plants—due to the fundamental event of endosymbiosis at the origin of the chloroplast [17]—a closer functional similarity between cyanobacterial and plant PCSs might be postulated and explored by more sensitive analytical techniques than those previously employed.

If we are to delve into the past to understand the present, we feel that it is appropriate to focus not only on cyanobacteria, but also on early plants that can give primeval information, such as liverworts (Marchantiophyta), regarded as the oldest living land plants [18,19]. To this end, the relatively simple and primitive traits of liverworts can be extremely important for investigating ancient functional and molecular characters, which mark the evolution of more recent land plants.

Therefore, in this paper, we have focused our attention on two photoautotrophic model-organisms, namely the cyanobacterium *Geitlerinema* sp. strain PCC 7407 and the liverwort *Marchantia polymorpha* L.

*Geitlerinema* sp. PCC 7407 [20] is a filamentous freshwater/terrestrial cyanobacterium, of particular importance due to the broad response mechanisms that it may activate as a result of different stresses [21]. *Geitlerinema* sp. can grow in the same natural environments as *Marchantia* sp. [22] and also presents the considerable advantage of being relatively simply cultivated in axenic conditions, in a growth chamber. Most importantly, research on the entire genome of *Geitlerinema* sp. PCC 7407 has identified a PCS gene that codifies a putative protein of 401 amino acids, fairly similar to plant PCSs (https://www.ncbi.nlm.nih.gov/assembly/GCF_000317045.1/) [20]. However, the PCS in *Geitlerinema* sp. PCC 7407 has never been characterised from a functional standpoint.

*M. polymorpha* is an extant thalloid liverwort with a key role in plant phylogeny [23,24]. In recent times, the development of transformation techniques and the sequencing of the entire genome [24,25] have rendered *M. polymorpha* a fundamental model-organism for functional, molecular, and evolutionary studies on land plants. In addition to the fact that *M. polymorpha* gametophytes can be axenically cultivated in a growth chamber, they also offer a number of other advantages, such as a short life cycle, a relatively small genome size (ca. 280 Mb), a haploid number of chromosome sets, and ease in propagation and crossing [24]. *M. polymorpha* currently includes three subspecies: (1) subsp. *polymorpha*; (2) subsp. *montivagans*; and (3) subsp. *ruderalis*. The latter corresponds to *M. polymorpha sensu stricto* [23], i.e., the taxon in which nuclear and organellar genomes have been entirely sequenced [24]. *M. polymorpha* has a high capacity for acquiring metals and may therefore provide a valuable tool for metal biomonitoring [26,27]. For all these reasons, in-depth investigations on *M. polymorpha* PCS can provide relevant information on functional aspects of this enzyme in plants and allow useful comparisons with the cyanobacterial PCS.

Therefore, in this work we performed a comparative functional characterization of the PCSs in the cyanobacterium *Geitlerinema* sp. strain PCC 7407 and in the gametophytes of the liverwort *M. polymorpha* subsp. *ruderalis*. The experimental approach was aimed at investigating the enzymatic activities of the respective PCSs in vitro and the production of PCn *in vivo*, in response to various Cd concentrations and exposure times. At the same time, we carried out a comparative quantification of the GSH levels in both organisms, taking into account that this fundamental thiol-tripeptide can exert direct and indirect effects in contrasting detrimental effects of Cd stress [28]. The experiments reported here will accordingly expand the current knowledge in the field of metal responses in cyanobacteria and early land plants.

## 2. Results

### 2.1. Phytochelatin Synthases from M. polymorpha and Geitlerinema sp. PCC 7407 are Constitutively Expressed and Produce Phytochelatins in vitro

The in vitro assays of PCSs from *Geitlerinema* sp. PCC 7407 cultures and *M. polymorpha* gametophytes revealed that both prokaryotic and eukaryotic PCSs are constitutive enzymes able to produce, even in the complete absence of Cd, traces of PC_2-4_ (only PC_2_ in *Geitlerinema* sp. PCC 7407) (Table 1). 

Under a Cd concentration of 100 µM for 90 min (see paragraph 4.2 in the M&M section for details), *M. polymorpha* PCS produced in vitro not only relatively high levels of PC_2_, but also detectable levels of PC_3_ and PC_4_ (Table 1). Specifically, the PC_2_/PC_3_, PC_2_/PC_4_, and PC_3_/PC_4_ ratios were equal to ca. 23, 31, and 1.3, respectively. Overall, the PCS activity measured in vitro under 100 µM Cd increased by ca. 77% compared to Cd-untreated controls (Table 1).

With the same Cd concentration (100 µM, the in vitro assay of the PCS from *Geitlerinema* sp. PCC 7407 under identical experimental conditions resulted in a high increase in the enzyme activity compared with controls. PCS activation led to PC_2_, PC_3_, and PC_4_ production at trace levels (Table 1), with PC_2_/PC_3_, PC_2_/PC_4_, and PC_3_/PC_4_ ratios of ca. 24, 32, and 1.3, respectively, and thus almost identical to those of *M. polymorpha*. Overall, the cyanobacterium PCS activity quantified in vitro under 100 µM Cd increased by ca. 91% compared to Cd-untreated controls (Table 1).

Finally, following the same Cd treatment in vitro, the overall PCn level measured in *Geitlerinema* sp. PCC 7407 was ca. 2.2% of the PCn quantified in *M. polymorpha*, assayed in identical conditions. In turn, the in vitro PCn levels quantified in *Geitlerinema* sp. PCC 7407 and *M. polymorpha* were, respectively, equal to approx. 0.1% and 4.9% of those of *Arabidopsis thaliana* treated with an identical concentration of Cd and tested by the same procedure (Table 1).

### 2.2. Cadmium Induces Phytochelatin Synthesis in vivo both in M. polymorpha and Geitlerinema sp. PCC 7407

**Figure 1:**
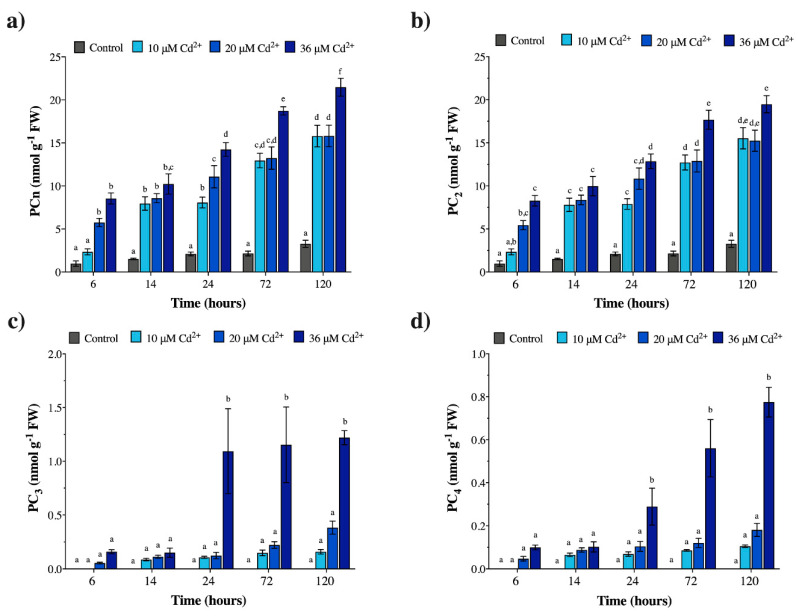
Phytochelatin (PCn) concentrations (nmol g^−1^ FW) in *M. polymorpha* gametophytes exposed *in vivo* to 0 (control), 10, 20, and 36 µM Cd. Sampling times equal to 6, 14, 24, 72, and 120 h of Cd exposure. (**a**) Total PCn; (**b**) PC_2_; (**c**) PC_3_; (**d**) PC_4_. Values are the mean ± SE; n = 5. Different letters indicate significant differences at *p* < 0.05 (two-way ANOVA, followed by Tukey’s post-hoc test).

**Figure 2:**
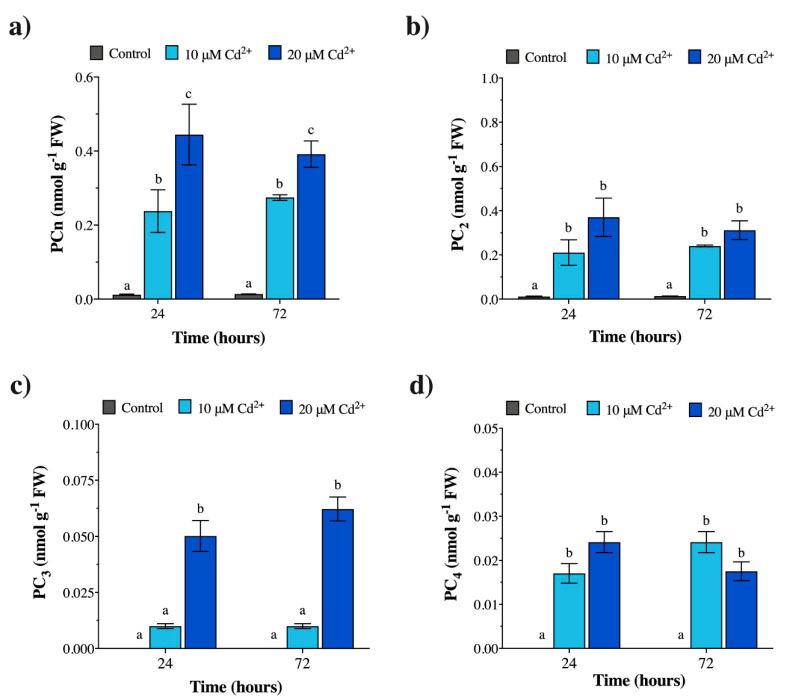
Phytochelatin (PCn) concentrations (nmol g^−1^ FW) in *Geitlerinema* sp. PCC 7407 cultures exposed *in vivo* to 0 (control), 10, and 20 µM Cd. Sampling times equal to 24 and 72 h of Cd exposure. (**a**) Total PCn; (**b**) PC_2_; (**c**) PC_3_; (**d**) PC_4_. Values are the mean ± SE; n = 5. Different letters indicate significant differences at *p* < 0.05 (two-way ANOVA, followed by Tukey’s post-hoc test).

The *in vivo* quantification of PCn from *Geitlerinema* sp. PCC 7407 cultures and *M. polymorpha* gametophytes confirmed the results of the PCS in vitro activity, in that PC_2_ was the only PCn oligomer always present in both organisms in the absence of Cd (controls), at all exposure times (Figure 1b and Figure 2b; Table S1); in fact, PC_3_ and PC_4_ were never detected in the controls (Figure 1c,d and Figure 2c,d; Table S1). 

With regard to *M. polymorpha*, following rises in Cd concentrations and exposure times, the PCn produced *in vivo* gradually increased (Figure 1a; Table S1). A representative HPLC-ESI-MS-MS chromatogram of GSH, PC_2_, PC_3_, and PC_4_ produced by *M. polymorpha* under 20 µM Cd for 72 h is shown in Figure S1a. In general, the *in vivo* analysis showed that the overall levels of PCn produced by the liverwort gametophytes were, on average, rather low (Figure 1a; Table S1). Moreover, as with the in vitro assays, the increases were essentially caused by rises in PC_2_ levels at all Cd concentrations and exposure times (Figure 1b; Table S1), as well as by the increase in PC_3_ and PC_4_ at the highest Cd concentration (36 µM) and longest exposure times (24, 72, and 120 h) (Figure 1c,d; Table S1). In particular, the PC_2_/PC_3_ and PC_2_/PC_4_ ratios remained extremely high at the lowest Cd concentrations (e.g., PC_2_/PC_3_ ratios equal to ca. 73–97 at 10 µM Cd, and raised up to ca. 99 at 20 µM Cd; PC_2_/PC_4_ ratios up to ca. 148 at 10 µM Cd, and ca. 84–115 at 20 µM Cd; Table S1), but dropped to ca. 16–35 as the metal concentration and the exposure times increased (e.g., 36 µM Cd at 72–120 h; Table S1). The PC_3_/PC_4_ ratio, on the other hand, maintained a generally constant trend through various Cd exposure times and 10–20 µM metal concentrations (ca. 1.2–2.1; Table S1), with perhaps an upward trend under 36 µM Cd at 24–72 h (ca. 1.6–3.8; Table S1).

In the case of *Geitlerinema* sp. PCC 7407, following Cd treatments at all exposure times, the *in vivo* PCn levels drastically increased with respect to the controls (Figure 2a; Table S1). In general, as also shown by the in vitro assay, the overall levels of PCn induced by Cd in *Geitlerinema* sp. were, on average, extremely low (Figure 2a; Table S1), in that they were at least 35–40-fold lower than those produced by *M. polymorpha* at the same Cd concentrations and exposure times (10–20 µM Cd for 24–72 h) (Figure 2a; Table S1). Specifically, at 24–72 h, 20 µM Cd induced amounts of PC_2_ and PC_4_ not significantly different from those induced at 10 µM Cd; on the contrary, at the highest concentration of 20 µM Cd, PC_3_ was produced in higher amounts compared with 10 µM Cd (Figure 2b–d; Table S1). A representative HPLC-ESI-MS-MS chromatogram of GSH, PC_2_, PC_3_, and PC_4_ produced by *Geitlerinema* sp. PCC 7407 under 20 µM Cd for 72 h is shown in Figure S1b. In terms of the mutual ratios between the PCn produced, under 10 µM Cd and at exposure-times of 24–72 h, the PC_2_/PC_3_ ratio was quite high (e.g., equal to ca. 21–24), but dropped to ca. 5–7 as the metal concentration increased to 20 µM Cd (Table S1). On the contrary, at the same exposure times, the PC_2_/PC_4_ ratio exhibited a reversed trend, with values of ca. 10–12 at 10 µM Cd and ca. 15–18 at 20 µM Cd (Table S1). Similarly, the PC_3_/PC_4_ ratio varied from ca. 0.4–0.6 at the lowest Cd concentration to ca. 2–3.5 at the highest concentration (Table S1).

### 2.3. Cadmium Exposure Increases GSH Levels in M. polymorpha, but Causes the Opposite Effect in Geitlerinema sp. PCC 7407

**Figure 3:**
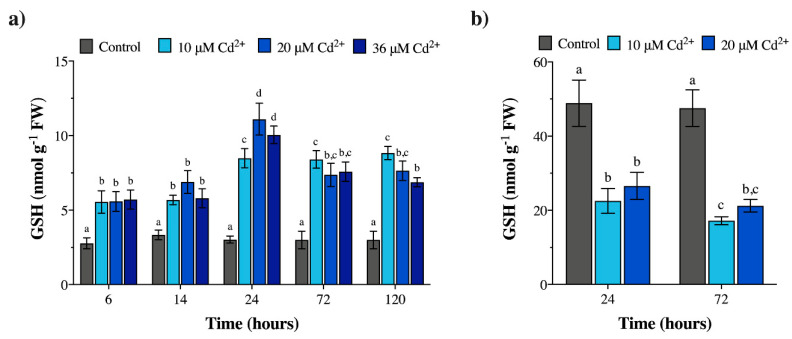
Glutathione (GSH) concentrations (nmol g^−1^ FW) in (**a**) *M. polymorpha* exposed to 0 (control), 10, 20, and 36 µM Cd for 6, 14, 24, 72, and 120 h; (**b**) *Geitlerinema* sp. PCC 7407 exposed to 0 (control), 10, and 20 µM Cd for 24 and 72 h. Values are the mean ± SE; n = 5. Different letters indicate significant differences at *p* < 0.05 (two-way ANOVA, followed by Tukey’s post-hoc test).

With regard to GSH, in control gametophytes of *M. polymorpha*, low and constant levels of this thiol-tripeptide were detected over time (Figure 3a; Table S1). In Cd-exposed samples, higher GSH levels than those of controls were measured, with the highest amounts detected after 24 h under 20–36 µM Cd (Figure 3a; Table S1). At all Cd concentrations at 6–14 h exposure times, the GSH ratios between Cd-treated samples and respective controls were fairly constant (ca. 1.7–2; Table S1), but increased at 24–72–120 h (ca. 2.3–2.9; Table S1), with a particular rise up to ca. 3.3–3.7 at 24 h with 20 and 36 µM Cd (Table S1).

Unlike *M. polymorpha*, in control samples of *Geitlerinema* sp. PCC 7407, at all concentrations/exposure times, much higher GSH levels than those of Cd-exposed samples were quantified (Figure 3b; Table S1). The GSH ratios between Cd-treated samples and respective controls were constant over time, with values ranging around 0.4–0.5 (Suppl. Table S1).

## 3. Discussion

Based on the results obtained in this work, five main points can be emphasized: (1) The PCS enzyme is constitutively expressed in both the liverwort (gametophytes of *M. polymorpha* subsp. *ruderalis*) and the cyanobacterium (cultures of *Geitlerinema* sp. strain PCC 7407); (2) in the absence of Cd, the PCSs of both organisms are able to produce a basal level of PCn; (3) in the presence of Cd at all concentrations, the PCSs from the two organisms are promptly activated and produce PCn, namely PC_2_, PC_3_, and PC_4_; (4) in both organisms, the increasing Cd concentrations and exposure times result in an increase in PCn levels, particularly PC_2_, PC_3_, and PC_4_ in the liverwort and PC_3_ in the cyanobacterium; (5) the constitutive levels of GSH in the liverwort, relative to those normally found in higher plants, are quite low, but increase significantly under Cd treatments. Conversely, the constitutive levels of GSH in the cyanobacterium are high, but the Cd treatments cut the levels by about 50%.

With regard to points (1), (2), and (3), although the constitutive expression of the PCS in *M. polymorpha* gametophytes had already been hypothesized in former work [4], it had not been demonstrated in full. Accordingly, the present study filled this gap, yielding specific data on the activity of *M. polymorpha* PCS in the absence/presence of Cd. The PCS activity data reported here for *M. polymorpha* gametophytes are in fact wholly consistent with those of Petraglia et al. (2014) and Degola et al. (2014) [4,29] for gametophytes of other liverwort species, i.e., *Conocephalum conicum*, *Pellia epiphylla*, *Radula complanata*, *Aneura pinguis*, *Scapania undulata*, and *Lunularia cruciata*, all assayed under fully comparable experimental conditions. Therefore, *M. polymorpha*, as well as other liverworts, possesses a constitutive PCS which is able to produce PCn by means of its transpeptidasic activity.

Concerning the cyanobacterium *Geitlerinema* sp. PCC 7407, the presence of a putative PCS of 401 amino acids with a fair homology with plant PCSs had already been hypothesized (https://www.ncbi.nlm.nih.gov/assembly/GCF_000317045.1/) [20], but functional investigations in this direction were lacking. Therefore, our work demonstrates not only that *Geitlerinema* sp. PCC 7407 possesses a constitutive PCS, but also that this enzyme displays manifest Cd-induced transpeptidasic activity. The very few experiments performed in this field in cyanobacteria—carried out exclusively in *Nostoc* sp. PCC 7120 [12,14,15]—have shown that the PCS only acts as a peptidase, with GSH-degrading activity exclusively aimed at regulating the catabolism of GSH-conjugates [13,14]. Indeed, in Harada et al. (2004) [14], no PCn synthesis or differences in PCS activity in the presence/absence of Cd were detected. Only Tsuji et al. (2004; 2005) [12,15] have demonstrated that the PCS from *Nostoc* sp. PCC 7120 possesses extremely weak transpeptidasic activity, only leading to the formation of very low amounts of PC_2_, but with no differences in PC_2_ levels between the presence and absence of Cd. 

Thus, our work demonstrates that the widely held notion that all cyanobacteria are basically unable to synthesize PCn and do not possess PCSs displaying full transpeptidasic activity is incorrect, or at least not transposable to all cyanobacteria. In this sense, it is probable that the development of modern experimental protocols with a very high sensitivity, such as those employed in our paper and based on the methodologies validated in a recent work [30], helped to obtain qualitative and quantitative data, which would have proven difficult to achieve a few years ago. 

In any case, on the other hand, it is true that the in vitro PCS activities measured in *M. polymorpha* and, above all, in *Geitlerinema* sp. PCC 7407, were (very) low—only approx. 4.9% and 0.1%, respectively, of those of *A. thaliana* PCS assayed in identical conditions. It should also be considered that the in vitro PCS activities of two lycophytes (i.e., *Huperzia selago* and *Selaginella denticulata*) were about 10–13% of the in vitro activity of the *A. thaliana* PCS [4], thus suggesting a trend toward higher PCS activities in the evolutionary history of plants. 

With reference to the above point (4), increases in Cd concentrations and exposure times in *M. polymorpha* not only increased PC_2_ levels, but also PC_3_ and PC_4_ levels, which, due to the higher number of thiol groups, potentially raised the Cd-chelating power [31], and thus the effectiveness in metal detoxification. Indeed, as far as we know, only in the liverworts *C. conicum* and *L. cruciata* PCn up to PC_4_ were detected [4,29], whereas in all the other species assayed so far, the PCn production was limited to only PC_2_ (just in one case to PC_2_ and PC_3_) [4]. Additionally, the data reported here confirm a similar behavior in *M. polymorpha* and higher plants: the more Cd is supplied (and the longer exposure lasts, within limits), the higher levels of PCn are synthesized [32]. A positive correlation between the duration/levels of Cd exposure and degree of PCn oligomerization in higher plants has in fact been reported [33,34]. Concerning *Geitlerinema* sp. PCC 7407, the increase in the degree of oligomerization of PCn over time in the presence of Cd regarded only PC_3_, and this response is perhaps consistent with the presence of a PCS with low transpeptidasic activity.

With regard to GSH (point 5 of the above outline), this thiol-tripeptide is a ubiquitous molecule in all organisms and is involved in a plethora of cellular mechanisms, mainly with functions as an antioxidant and regulator of redox homeostatic processes [35,36]. In *M. polymorpha*, the GSH amounts found in controls and in Cd-exposed samples were wholly comparable to those quantified in other liverworts [4,29], with the sole exception of *P. epiphylla*, which is the only species with GSH levels 6–60 times higher than those of other liverworts [4], *M. polymorpha* included. Interestingly, both in the liverworts studied previously (except *P. epiphylla*) and in *M. polymorpha*, the exposure to Cd resulted in marked increases in GSH amounts, possibly reflecting re-synthesis by the enzyme glutathione synthase and/or the reduction of oxidized glutathione (GSSG) by the NADPH-dependent glutathione reductase.

Unlike *M. polymorpha*, the cyanobacterium *Geitlerinema* sp. PCC 7407 showed high constitutive levels of GSH. In general, elevated amounts of GSH are measured in cyanobacteria [37,38]. Indeed, a study concerning the evolution of GSH in photoautotrophic microorganisms reported high levels of this thiol-tripeptide in nine cyanobacterial strains [39]. In filamentous cyanobacteria, such as *Anabaena laxa* and *Nostoc muscorum*, the constitutive GSH levels detected [38] were very similar to those reported here for *Geitlerinema* sp. PCC 7407. As with plants, the main role played by GSH in cyanobacteria is antioxidant protection [37,40]. The significant decrease in GSH levels observed in the present study in *Geitlerinema* sp. PCC 7407, following exposure to Cd, suggests that this cyanobacterium may use GSH as a direct ligand for Cd sequestration, in addition to as a precursor for PCn synthesis. This hypothesis is consistent with the low levels of Cd-induced PCn detected in the cyanobacterium. Vivares et al., (2005) [1] recorded interesting results with the PCS from *Nostoc* sp. PCC 7120; no PCn were in fact produced in this organism, possibly mechanistically resulting from insufficient space to accommodate adequate amounts of the substrate GSH in the active site of the enzyme. Assuming the truthfulness of this hypothesis also for *Geitlerinema* sp. PCC 7407, it is reasonable to suppose that the GSH not depleted by the cyanobacterial PCS for synthesizing PCn is instead employed as a direct Cd chelator.

In view of the above, from our comparative analysis between the liverwort *M. polymorpha* and the cyanobacterium *Geitlerinema sp.* PCC 7407, it follows that both eukaryotic and prokaryotic PCSs appear to differ less in functional terms than previously thought. From an evolutionary point of view, various studies postulate that high heavy metal concentrations present in paleo-environments may have posed severe problems for ancient life forms, including cyanobacteria and early plants ([16,41] and reference therein). Therefore, under selection pressure, these organisms might have evolved/improved similar mechanisms to efficiently cope with metals. The transpeptidasic activity of the PCS enzymes and the consequent PCn production might have positively contributed to these processes.

## 4. Materials and Methods 

### 4.1. Growth Conditions and Experimental Set-Up

In vitro cultures of *Marchantia polymorpha* L. subsp. *ruderalis* Bischl. and Boisselier-Dubayle (Marchantiales, Marchantiophyta) female gametophytes (Cam2–Cambridge-2 wild type, University of Cambridge, UK) were grown in Petri dishes starting from the axenic cultivation of gemmae (from “gemmae cups”), in half-strength Murashige and Skoog (MS ½) medium (Duchefa Biochemie, Haarlem, The Netherlands), supplemented with 0.8% (w/v) sucrose (Duchefa Biochemie) and 0.7% (w/v) agar (Duchefa Biochemie). The MS ½ pH was adjusted to 5.7 with KOH. The culture conditions were set at a 16:8 h light/dark cycle, 19 ± 1 °C, and a photosynthetic photon flux density of 60 µmol m^−2^ s^−1^, with a 60% relative humidity. After a two-week cultivation of gemmae, the derived axenic gametophytes were transferred into fresh Petri dishes for another two weeks. The four-week-old gametophytes were then moved into sterile pots filled with liquid MS ½ medium (as described above, without agar) for a further two weeks. Thereafter, the six-week-old gametophytes (about 0.8 g FW) were independently placed in different treatment conditions: control (MS ½ without Cd, pH 5.7) and Cd-exposed (MS ½ with Cd, pH 5.7, at 10, 20, or 36 μM, provided as CdSO_4_ for 6, 14, 24, 72, and 120 h). After all treatments, control and Cd-exposed gametophytes were gently blotted dry with filter paper, split into 100 mg FW samples, frozen in liquid nitrogen, and stored at −80 °C until use. Five biological replicates for each sampling time were performed.

Axenic cultures of *Geitlerinema* sp. strain PCC 7407 (Pasteur Culture Collection, Paris, France) (Oscillatoriales, Cyanobacteria) were grown in BG11 medium [42], at a pH adjusted to 7.1 with HCl. The cultures were set up in batch in 400 ml flasks and kept under a 12:12 h light/dark cycle at 18 ± 1 °C and a photosynthetic photon flux density of 20 µmol m^−2^ s^−1^. During the exponential growth phase (approx. reached in 10 days of cultivation, with an FW of ca. 20 mg ml^−1^), each culture was independently exposed to different treatments: control (BG11 medium without Cd, pH 7.1) and Cd-exposed (BG11 medium, pH 7.1, with 10, or 20 µM Cd-provided as CdSO_4_ for 24 and 72 h). The Cd concentration of 36 µM and the exposure time of 120 h were not employed, due to the toxicity recorded in preliminary tests (Erika Bellini, unpublished). Samples of 100 mg FW were then harvested through centrifugation at 24,000× *g* for 10 min (Heraeus SEPATECH, Megafuge 1.0, Thermo Fisher Scientific, Waltham, MA, USA), quickly frozen in liquid nitrogen, and stored at −80 °C until use. Five biological replicates for each sampling time were performed.

### 4.2. Phytochelatin Synthase Activity Assays

The PCS activity was assayed in 200 mg of FW material (Cd-untreated) from *M. polymorpha* gametophytes and *Geitlerinema* sp. PCC 7407 culture biomass, as described in Petraglia et al. (2014) [4], with some slight modifications. Briefly, each sample was placed in a 2 ml Eppendorf tube, supplied with liquid nitrogen, and ground to a powder with a mixer mill (MM200, Retch, Haan, Germany), using two agate grinding balls (5 mm diameter) to facilitate mechanical cell lysis at a vibrational frequency of 30 Hz for 1 min. The powder was then mixed to 700 µl of extraction buffer and homogenized for 1 min with another cycle of mixer mill shaking. The homogenized samples were centrifuged twice at 13,000× *g* (Hermle centrifuge, Z 300 K, Wehingen, Germany) at 4 °C for 10 min, and 400 µl of the supernatants was added to 100 μl of the reaction buffer, containing 100 µM Cd (provided as CdSO_4_) and the protease inhibitor cocktail ‘complete mini EDTA-free’ (Roche Italia, Milan, Italy). After an incubation time of 90 min at 35 °C, the reaction was terminated with 125 µl of 20% trichloroacetic acid and the measurement of PCS activity was immediately performed by HPLC-ESI-MS-MS, as described in point 4.3. As a reference organism for PCS activities, *Arabidopsis thaliana* 21-day-old in vitro sterile plants were tested under the same conditions employed for *M. polymorpha* gametophytes and *Geitlerinema* sp. PCC 7407 cultures. The PCS activities were expressed as pmol PCn g^−1^ FW min^−1^. Five biological replicates were performed.

### 4.3. Extraction, Detection, and Quantification of Thiol-Peptides 

Control and Cd-treated samples (100 mg FW) of *M. polymorpha* gametophytes and *Geitlerinema* sp. PCC 7407 culture biomass, previously stored at −80 °C, were extracted according to Bellini et al. (2019) [30]. Briefly, each sample was supplied with liquid nitrogen and ground to a powder with a mixer mill (MM200, Retch, Haan, Germany), using two agate grinding balls (5 mm diameter) to facilitate mechanical cell lysis at a vibrational frequency of 30 Hz for 1 min. Then, the extraction buffer (300 µl for *M. polymorpha* samples and 200 µl for *Geitlerinema* sp. PCC 7407 samples) was added to each sample, which was spiked with (glycine-^13^C_2_,^15^N)-labeled GSH and PC_2_ internal standards (each ISs at a concentration of 200 ng ml^−1^). Powder was re-suspended using a vortex mixer for 30 s and homogenized again using the mixer mill for 1 min at 30 Hz. The suspension was then vortexed for 30 s, put in ice for 15 min, and vortexed for 5 min. The extract was sedimented by centrifugation at 13,000× *g* (Hermle, Z 300 K, Wehingen, Germany) at 4 °C for 20 min, and the supernatants filtered through Minisart RC4 0.45 µm filters (Sartorius, Goettingen, Germany) and then stored at −80 °C.

HPLC-ESI-MS-MS analyses were performed by an instrument layout consisting of an Agilent 1290 Infinity UHPLC (Santa Clara, CA, USA), inclusive of a thermostated autosampler, a binary pump, and a column oven, coupled to an AB Sciex (Concord, ON, Canada) API 4000 triple quadrupole mass spectrometer, equipped with a Turbo-V Ion spray source. A ten-port switching valve (Valco Instruments Co. Inc., Huston, TX, USA) was used as a diverter valve (Concord, ON, Canada). Chromatographic separation was performed by a reverse-phase Phenomenex (Torrance, CA, USA) Kinetex 2.6 µm XB-C18 100 Å, 100 × 3 mm HPLC column, protected by a C18 3 mm ID security guard ULTRA cartridge. All of the above analyses and the quantification of thiol-peptides were performed following the procedures detailed in Bellini et al. (2019) [30]. System control, data acquisition, and processing were carried out by AB Sciex Analyst^®^ version 1.6.3 software.

### 4.4. Statistical Analyses

Data were analyzed by the Graph-Pad Prism 8.2.1 program (GraphPad Software Inc., Sand Diego, CA, USA) and reported as mean ± SE (standard error). The threshold of statistical significance was set at *p* < 0.05. Data related to the in vitro PCS activity were analysed by the Mann–Whitney test, whereas data on PCn and GSH *in vivo* production were analysed by two-way ANOVA, followed by Tukey’s multiple comparison post-hoc test.

## 5. Conclusions

In vitro and *in vivo* assays of PCSs from axenic *M. polymorpha* gametophytes and axenic *Geitlerinema* sp. PCC 7407 cultures revealed first that eukaryotic and prokaryotic PCSs are constitutive enzymes. Second, PCSs in both organisms are activated by Cd and are able to synthesize PCn up to PC_4_. Therefore, eukaryotic and prokaryotic PCSs appear to differ less in functional terms than previously thought. 

## Figures and Tables

**Table 1:** In vitro activity assay of phytochelatin synthases (PCSs) from *Marchantia polymorpha* gametophytes and *Geitlerinema* sp. PCC 7407 cultures, either without Cd (control) or with the addition of 100 µM Cd. The assay was carried out at 35 °C for 90 min in the proper reaction buffer (see M&M). PCS activity (mean ± SE) is expressed as pmol PCn g^−1^ FW min^−1^; n = 5. Different letters indicate significant differences between PCS activity in control and in 100 µM Cd (Mann–Whitney test, *p* < 0.05). The increase (%) in PCS activity in the presence of 100 µM Cd, compared with the respective control, is also given, by setting controls equal to 0% in enzyme activity and expressing the Cd-increased activity in %. The percentage of PCS activity relative to that of *A. thaliana* PCS (equal to 1187.80 ± 14.27 pmol PCn g^−1^ FW min^−1^, fixed as 100%), assayed with the same Cd concentration and buffer conditions, is also indicated.

	PCS Activity Without Cd^2+^ (Control) (pmol PCn g^−1^ FW min^−1^)	PCS Activity With 100 µM Cd^2+^ (pmol PCn g^−1^ FW min^−1^)	Increase in PCS Activity with 100 µM Cd^2+^ Compared with Controls (%)	Relative PCS Activity (% of *A. thaliana* PCS Activity)
*M. polymorpha*				
PC_2_	16.94 ± 1.65 a	70.88 ± 2.32 b	76.3 ± 1.6	
PC_3_	0.60 ± 0.06 a	3.08 ± 0.22 b	80.6 ± 1.2	
PC_4_	0.39 ± 0.05 a	2.29 ± 0.32 b	82.5 ± 1.7	
Total PCn	17.94 ± 1.62 a	76.26 ± 2.18 b	76.7 ± 1.5	4.91
*Geitlerinema* sp. PCC 7407				
PC_2_	0.156 ± 0.01 a	1.559 ± 0.01 b	90.0 ± 0.9	
PC_3_	0.000 ± 0.00 a	0.064 ± 0.01 b	100.0 ± 0.0	
PC_4_	0.000 ± 0.00 a	0.049 ± 0.01 b	100.0 ± 0.0	
Total PCn	0.156 ± 0.01 a	1.672 ± 0.01 b	90.7 ± 0.9	0.13

## Supplementary Materials

The following are available online at https://www.mdpi.com/2223-7747/9/7/914/s1, Figure S1: Representative HPLC-ESI-MS-MS chromatograms of (a) Marchantia polymorpha gametophytes and (b) *Geitlerinema* sp. PCC 7407 cultures, treated with 20 µM Cd for 72 h; Table S1: GSH and phytochelatin amounts and ratios in Marchantia polymorpha and *Geitlerinema* sp. strain PCC 7407. Values are the mean ± SE.
