# Supplementary material for: Eukaryotic and Prokaryotic Phytochelatin Synthases Differ Less in Functional Terms Than Previously Thought: A Comparative Analysis of Marchantia polymorpha and Geitlerinema sp. PCC 7407
Source: Plants (Basel). 2020 Jul 20;9(7):914. doi: 10.3390/plants9070914 (PMC7411734; doi:10.3390/plants9070914)
Supplement: Supplementary file 1 [file plants-09-00914-s001.zip › plants-854809-revised1-sup/supplementary files/Table S1 PLANTS.docx]

**Table 1.** GSH and phytochelatin amounts and ratios in *Marchantia polymorpha* and *Geitlerinema* sp. strain PCC 7407. Values are mean ± SE.

|  | **Time (hours)** | **GSH**  **(nmol g^-1^ FW)** | **GSH treated/GSH untreated** | **PC_2_**  **(nmol g^-1^ FW)** | **PC_3_**  **(nmol g^-1^ FW)** | **PC_4_**  **(nmol g^-1^ FW)** | **Total PCn**  **(nmol g^-1^ FW)** | **PC_2_/PC_3_** | **PC_2_/PC_4_** | **PC_3_/PC_4_** |
| --- | --- | --- | --- | --- | --- | --- | --- | --- | --- | --- |
| *M. polymorpha* |  |  |  |  |  |  |  |  |  |  |
| Control | 6 | 2.769 ± 0.372 |  | 0.972 ± 0.314 | 0.000 ± 0.000 | 0.000 ± 0.000 | 0.972 ± 0.314 |  |  |  |
|  | 14 | 3.341 ± 0.327 |  | 1.509 ± 0.090 | 0.000 ± 0.000 | 0.000 ± 0.000 | 1.509 ± 0.090 |  |  |  |
|  | 24 | 3.025 ± 0.236 |  | 2.103 ± 0.216 | 0.000 ± 0.000 | 0.000 ± 0.000 | 2.103 ± 0.216 |  |  |  |
|  | 72 | 2.995 ± 0.587 |  | 2.153 ± 0.274 | 0.000 ± 0.000 | 0.000 ± 0.000 | 2.153 ± 0.274 |  |  |  |
|  | 120 | 2.992 ± 0.592 |  | 3.274 ± 0.409 | 0.000 ± 0.000 | 0.000 ± 0.000 | 3.274 ± 0.409 |  |  |  |
| 10 µM Cd | 6 | 5.545 ± 0.752 | 2.00 ± 0.27 | 2.338 ± 0.347 | 0.000 ± 0.000 | 0.000 ± 0.000 | 2.338 ± 0.347 |  |  |  |
|  | 14 | 5.681 ± 0.311 | 1.70 ± 0.15 | 7.802 ± 0.770 | 0.086 ± 0.011 | 0.065 ± 0.008 | 7.954 ± 0.785 | 89.73 ± 0.23 | 119.24 ± 0.22 | 1.32 ± 0.26 |
|  | 24 | 8.485 ± 0.648 | 2.80 ± 0.15 | 7.894 ± 0.616 | 0.107 ± 0.009 | 0.069 ± 0.010 | 8.070 ± 0.631 | 73.47 ± 0.16 | 114.64 ± 0.22 | 1.56 ± 0.23 |
|  | 72 | 8.404 ± 0.591 | 2.80 ± 0.16 | 12.716 ± 0.871 | 0.148 ± 0.025 | 0.086 ± 0.003 | 12.950 ± 0.847 | 85.53 ± 0.24 | 148.24 ± 0.11 | 1.73 ± 0.22 |
|  | 120 | 8.834 ± 0.444 | 2.95 ± 0.25 | 15.527 ± 1.239 | 0.159 ± 0.021 | 0.105 ± 0.005 | 15.792 ± 1.255 | 97.48 ± 0.21 | 147.51 ± 0.13 | 1.51 ± 0.19 |
| 20 µM Cd | 6 | 5.582 ± 0.658 | 2.01 ± 0.25 | 5.441 ± 0.534 | 0.054 ± 0.007 | 0.047 ± 0.010 | 5.750 ± 0.480 | 99.51 ± 0.24 | 115.26 ± 0.32 | 1.16 ± 0.37 |
|  | 14 | 6.886 ± 0.758 | 2.06 ± 0.21 | 8.373 ± 0.554 | 0.113 ± 0.014 | 0.087 ± 0.010 | 8.574 ± 0.529 | 74.01 ± 0.19 | 95.27 ± 0.18 | 1.29 ± 0.24 |
|  | 24 | 11.102 ± 1.069 | 3.67 ± 0.17 | 10.838 ± 1.239 | 0.123 ± 0.030 | 0.104 ± 0.023 | 11.066 ± 1.293 | 87.82 ± 0.36 | 103.65 ± 0.33 | 1.18 ± 0.47 |
|  | 72 | 7.366 ± 0.779 | 2.46 ± 0.30 | 12.890 ± 1.277 | 0.222 ± 0.030 | 0.120 ± 0.021 | 13.233 ± 1.298 | 57.99 ± 0.23 | 106.95 ± 0.28 | 1.84 ± 0.31 |
|  | 120 | 7.643 ± 0.652 | 2.55 ± 0.28 | 15.240 ± 1.233 | 0.383 ± 0.060 | 0.181 ± 0.030 | 15.805 ± 1.257 | 39.69 ± 0.23 | 84.09 ± 0.25 | 2.12 ± 0.32 |
| 36 µM Cd | 6 | 5.704 ± 0.643 | 2.06 ± 0.25 | 8.278 ± 0.611 | 0.160 ± 0.018 | 0.099 ± 0.011 | 8.537 ± 0.640 | 51.71 ± 0.18 | 83. 08 ± 0.18 | 1.61 ± 0.23 |
|  | 14 | 5.798 ± 0.629 | 1.73 ± 0.21 | 9.970 ± 1.128 | 0.150 ± 0.042 | 0.102 ± 0.024 | 10.223 ± 1.180 | 66.03 ± 0.39 | 97.38 ± 0.34 | 1.47 ± 0.51 |
|  | 24 | 10.049 ± 0.590 | 3.32 ± 0.14 | 12.856 ± 0.843 | 1.093 ± 0.395 | 0.289 ± 0.085 | 14.238 ± 0.798 | 11.75 ± 0.42 | 44.49 ± 0.36 | 3.78 ± 0.66 |
|  | 72 | 7.580 ± 0.653 | 2.53 ± 0.28 | 17.676 ± 1.100 | 1.153 ± 0.351 | 0.560 ± 0.133 | 18.724 ± 0.472 | 15.32 ± 0.36 | 31.54 ± 0.30 | 2.06 ± 0.54 |
|  | 120 | 6.872 ± 0.302 | 2.29 ± 0.24 | 19.477 ± 0.995 | 1.220 ± 0.065 | 0.775 ± 0.069 | 21.472 ± 1.038 | 15.96 ± 0.10 | 35.13 ± 0.14 | 1.57 ± 0.14 |
|  |  |  |  |  |  |  |  |  |  |  |
| *Geitlerinema* sp. PCC 7407 |  |  |  |  |  |  |  |  |  |  |
| Control | 24 | 48.863 ± 6.234 |  | 0.012 ± 0.001 | 0.000 ± 0.000 | 0.000 ± 0.000 | 0.012 ± 0.001 |  |  |  |
|  | 72 | 47.530 ± 4.927 |  | 0.014 ± 0.002 | 0.000 ± 0.000 | 0.000 ± 0.000 | 0.013 ± 0.001 |  |  |  |
| 10 µM Cd | 24 | 22.519 ± 3.352 | 0.46 ± 0.27 | 0.211 ± 0.058 | 0.009 ± 0.001 | 0.017 ± 0.002 | 0.237 ± 0.057 | 21.11 ± 0.38 | 12.37 ± 0.40 | 0.58 ± 0.24 |
|  | 72 | 17.186 ± 1.087 | 0.36 ± 0.17 | 0.240 ± 0.004 | 0.009 ± 0.001 | 0.024 ± 0.002 | 0.274 ± 0.007 | 24.09 ± 0.12 | 9.95 ± 0.12 | 0.41 ± 0.21 |
| 20 µM Cd | 24 | 26.552 ± 3.647 | 0.54 ± 0.26 | 0.370 ± 0.086 | 0.050 ± 0.007 | 0.024 ± 0.002 | 0.444 ± 0.081 | 7.37 ± 0.37 | 15.33 ± 0.33 | 2.08 ± 0.23 |
|  | 72 | 21.218 ± 1.688 | 0.44 ± 0.18 | 0.312 ± 0.042 | 0.062 ± 0.005 | 0.017 ± 0.002 | 0.391 ± 0.035 | 5.01 ± 0.22 | 17.79 ± 0.26 | 3.55 ± 0.21 |
