# Supplementary figures and images for: Eukaryotic and Prokaryotic Phytochelatin Synthases Differ Less in Functional Terms Than Previously Thought: A Comparative Analysis of Marchantia polymorpha and Geitlerinema sp. PCC 7407
Source: Plants (Basel). 2020 Jul 20;9(7):914. doi: 10.3390/plants9070914 (PMC7411734; doi:10.3390/plants9070914)

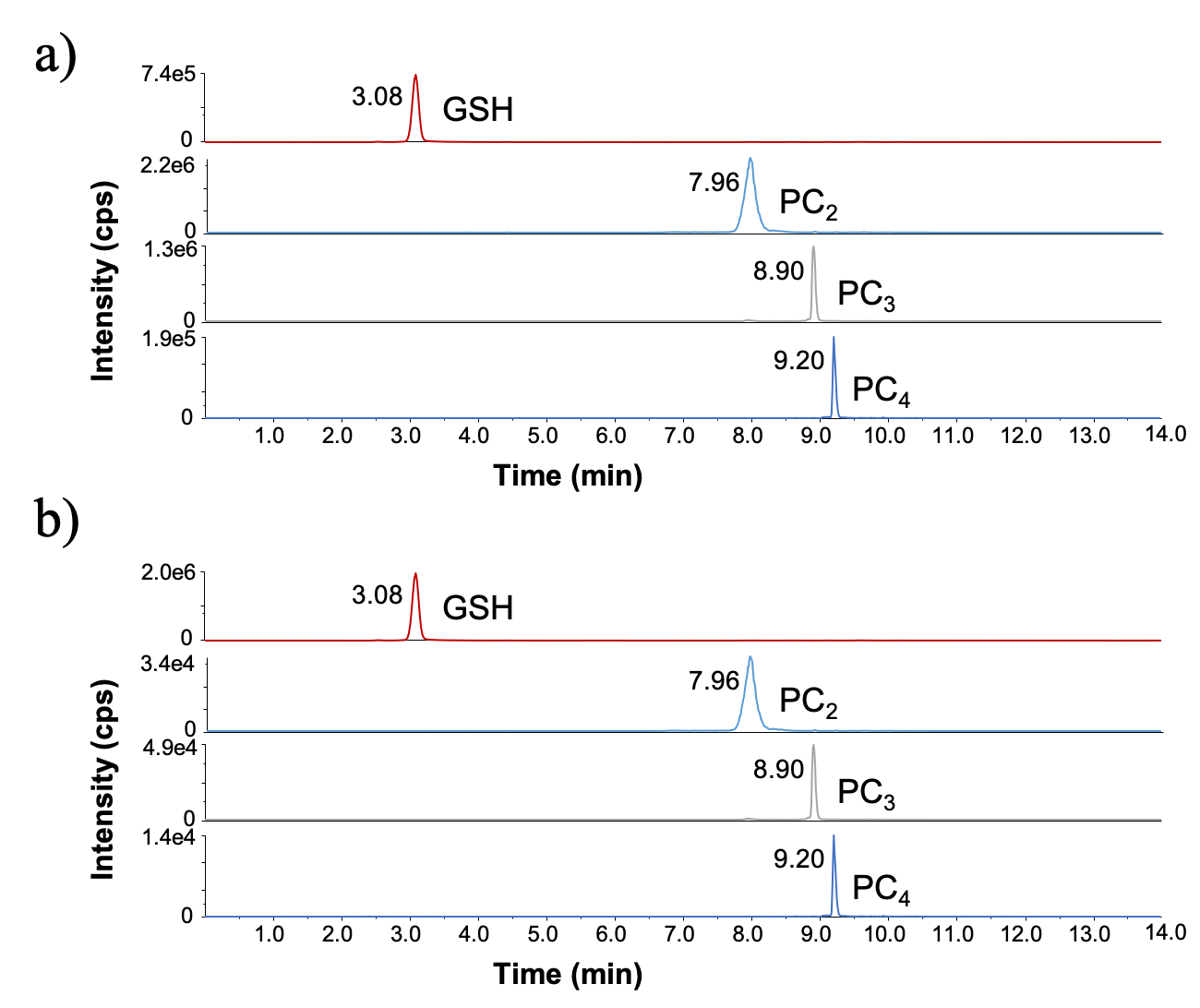

Supplement: Supplementary file 1 [file plants-09-00914-s001.zip › plants-854809-revised1-sup/supplementary files/Figure S1 PLANTS.png]
